# Proteomics and lipidomic analysis reveal dysregulated pathways associated with loss of sacsin

**DOI:** 10.3389/fnins.2024.1375299

**Published:** 2024-06-07

**Authors:** Daniele Galatolo, Silvia Rocchiccioli, Nicoletta Di Giorgi, Flavio Dal Canto, Giovanni Signore, Federica Morani, Elisa Ceccherini, Stefano Doccini, Filippo Maria Santorelli

**Affiliations:** ^1^Molecular Medicine, IRCCS Stella Maris Foundation, Pisa, Italy; ^2^Institute of Clinical Physiology, National Research Council, Pisa, Italy; ^3^Department of Biology, University of Pisa, Pisa, Italy; ^4^The BioRobotics Institute, Scuola Superiore Sant’Anna, Pisa, Italy

**Keywords:** autosomal recessive spastic ataxia of Charlevoix-Saguenay, ARSACS, SACS, fibroblasts, ceramides, diacylglycerols, Ca^2+^

## Abstract

**Introduction:**

Autosomal recessive spastic ataxia of Charlevoix-Saguenay (ARSACS) is a rare incurable neurodegenerative disease caused by mutations in the *SACS* gene, which codes for sacsin, a large protein involved in protein homeostasis, mitochondrial function, cytoskeletal dynamics, autophagy, cell adhesion and vesicle trafficking. However, the pathogenic mechanisms underlying sacsin dysfunction are still largely uncharacterized, and so attempts to develop therapies are still in the early stages.

**Methods:**

To achieve further understanding of how processes are altered by loss of sacsin, we used untargeted proteomics to compare protein profiles in ARSACS fibroblasts versus controls.

**Results:**

Our analyses confirmed the involvement of known biological pathways and also implicated calcium and lipid homeostasis in ARSACS skin fibroblasts, a finding further verified in SH-SY5Y *SACS*^–/–^ cells. Validation through mass spectrometry-based analysis and comparative quantification of lipids by LC-MS in fibroblasts revealed increased levels of ceramides coupled with a reduction of diacylglycerols.

**Discussion:**

In addition to confirming aberrant Ca^2+^ homeostasis in ARSACS, this study described abnormal lipid levels associated with loss of sacsin.

## 1 Introduction

Autosomal recessive spastic ataxia of Charlevoix-Saguenay (ARSACS, MIM #270550) is a rare, early-onset inherited neurological disorder characterized by degeneration of Purkinje cells and spinocerebellar connections, commonly leading to gait ataxia, spasticity, cerebellar atrophy, and peripheral neuropathy (Xiromerisiou et al., 2020). ARSACS is caused by mutations in *SACS* (Engert et al., 2000), a gene encoding sacsin whose “scaffold- like” and multidomain organization suggests involvement in protein quality control (Romano et al., 2013). Recent findings point to loss-of-function mechanism in ARSACS (Longo et al., 2021), and multiple *in vitro* and *in vivo* studies suggest that sacsin plays roles in mitochondrial dynamics (Girard et al., 2012; Criscuolo et al., 2015), cytoskeletal filament assembly and dynamics (Duncan et al., 2017; Louit et al., 2023), axonal development (Ady et al., 2018; Romano et al., 2022), and Ca^2+^ homeostasis (Del Bondio et al., 2023). Yet, the question of how mutant sacsin leads to disease status in patients with ARSACS remains to be answered.

Leveraging on previous RNA-Seq (Morani et al., 2019) and aptamer-based proteomic (Morani et al., 2021) studies, we observed that loss of sacsin impacts autophagic flux, bioenergetics, neuroinflammation, synaptogenesis, and engulfment of cells, mechanisms whose involvement was further confirmed by organelle-based quantitative proteomics in neuronal-like cells (Morani et al., 2022). Nonetheless, these new clues have not led to advances in our therapeutic approaches, which remain largely speculative or limited to preclinical models (Martinelli et al., 2020; Naef et al., 2021; Nethisinghe et al., 2021; Del Bondio et al., 2023; Toscano Márquez et al., 2023).

With the aim of broadening investigation of the consequences of sacsin loss of function, we performed a mass spectroscopy (MS)-based proteomic and lipidomic study in ARSACS fibroblasts. The results strengthen data about the key role of aberrant Ca^2+^ buffering in the disease, highlight disrupted lipid homeostasis in primary cells and neuronal-like models, and show that altered levels of ceramides and diacylglycerols may play a role in impaired cell signaling in ARSACS.

## 2 Materials and methods

### 2.1 Patients

This study was approved by the Tuscany Regional Pediatric Ethics Committee. Six patients with a clinical and genetic diagnosis of ARSACS (see Supplementary Table 1) were retrospectively recruited among the cohort of spastic-ataxic patients attending the research hospital IRCCS Stella Maris (Pisa, Italy). Patients were recruited irrespective of disease severity and genotype, but they had to have undergone at least two clinical evaluations within the past 12 months.

### 2.2 Cell cultures

Fibroblasts were isolated from skin biopsies of six affected patients (2 males, 4 females) and three healthy controls (1 male, 2 females). Control and *SACS*^–/–^ SH-SY5Y cells were previously generated and characterized (Morani et al., 2019). Cell lines were grown at 37°C with 5% CO_2_ in Dulbecco’s modified Eagle’s medium, supplemented with 10% fetal bovine serum, 4.5 g/L glucose, and 1% antibiotics/antimycotics. In accordance with the Declaration of Helsinki, all study participants gave their written informed consent to skin biopsy as a routine diagnostic procedure.

### 2.3 Mass spectrometry-based proteomics

Cell proteins extracted from six ARSACS patients (Pt 1-6) and three age-/sex-matched healthy controls were prepared for MS analysis as previously described (Di Giorgi et al., 2022). Chromatographic performances and time-of-flight (ToF) accuracy were occasionally evaluated using an intra-run injection of beta-galactosidase 100 fmol/l. Samples were analyzed in triplicate using an information-dependent acquisition (IDA) tandem MS method in a 5600 QTOF system (AB Sciex, Framingham, MA) (Di Giorgi et al., 2022). The Paragon Algorithm was used for false discovery rate (FDR) assessment (Shilov et al., 2007). The estimated number of false positive peptide identifications was then calculated to filter the true positive matches according to an FDR ≤ 5% threshold. Generated data were processed using the SWATH tool and Marker View software (AB Sciex) to extract the peak areas of all the identified peptides and proteins.

### 2.4 Proteomics data analysis

Protein abundances were calculated from the average MS peak intensity of controls and patients and then normalized on the basis of the mean total protein abundance per group. Differentially expressed proteins (DEPs) were identified as those with ≥ 2 unique peptides used for label-free quantitation at FDR < 0.01, and with a fold change (FC) ≥ 1.5. Statistical significance was set at *p* ≤ 0.05 with Student’s two-tailed *t*-test, and *p*-values were further corrected using the Benjamini-Hochberg. Proteomics experimental data were analyzed using R software (version 3.6.3)^1^ and DEPs underwent Gene Ontology (GO), Pathway, and Functional Analysis. GO analysis was conducted using the BiNGO plug-in (Maere et al., 2005) of the Cytoscape software (version 3.8.0).^2^ Enrichment analysis was performed using a hypergeometric test and resulting *p-*values were corrected using the Benjamini-Hochberg procedure. GO terms with a *p*-value < 0.01 were considered significant for the analysis. Biological Processes (BPs), Molecular Functions (MFs), and Cellular Components (CCs) were explored in three separate analyses. GO terms were clustered using the AutoAnnotate plug-in (GLay clustering algorithm) of the Cytoscape software. Afterwards, functional annotation analysis was performed using the Database for Annotation, Visualization, and Integrated Discovery (DAVID) Bioinformatics Resources 6.8 (Huang et al., 2009). Enrichment analysis was performed using a Fisher’s exact test followed by the Benjamini-Hochberg correction, setting significance at *p* < 0.01. Results were processed using the Functional Annotation Clustering tool. Enrichment scores were calculated on the basis of the cluster members’ *p*-values. The classification stringency adopted was high (similarity term overlap = 3, similarity threshold = 0.85, initial group membership = 3, final group membership = 3, multiple linkage threshold = 0.5). Final complete bioinformatic categorization of datasets and network analysis was carried out using Ingenuity Pathway Analysis (IPA™) (Qiagen, Hilden, Germany; IPA Winter Release—December 2020 and Spring Release—April 2022; version 73620684). A z-score value estimated the predicted activation or inhibition of a given biological function; only annotations with *p* < 0.05 and activation z-scores > 1.5 were included in the bioinformatics analysis.

### 2.5 Lipid droplets detection

To corroborate lipidomic data, we investigated the presence of lipid droplets in both ARSACS fibroblasts (Pt 2, Pt 4) and SH-SY5Y *SACS*^–/–^ cells. Cells were incubated overnight with lipid excess (200 μM oleic acid complexed to albumin, OA/BSA) and the size and number of lipid droplets were quantified upon BODIPY 493/503 staining (Thermo Fisher Scientific, Waltham, MA). Images were acquired using a Nikon Ti2-E inverted microscope. For data quantification, an unbiased method suitable for immunofluorescence staining was used to select and to count the droplet structures in each field (Doccini et al., 2022). Detection parameters were set to measure circular-like structures with sizes > 0.5 μm^2^ (fibroblasts) or 2 μm^2^ (SH-SY5Y). At least five fields from four different images of fibroblasts, 800 SH-SY5Y *SACS*^–/–^ cells (from 7 different fields), and 1400 SH-SY5Y control cells (from 10 different fields) were analyzed. Lipid droplets were measured and counted normalizing to the number of cells defined upon DAPI staining. Samples were analyzed in triplicate. Statistical analysis was performed using Prism version 7.04 (GraphPad Software, La Jolla, CA).

### 2.6 Intracellular calcium flux measurement

To further validate the involvement of calcium homeostasis in ARSACS, we assayed intracellular calcium flux using the Fluo-8 Calcium Flux Assay Kit (Abcam, Cambridge, United Kingdom) according to the manufacturer’s instructions. Fibroblasts from three healthy individuals and three patients with ARSACS (Pt2, Pt4, Pt6) were assayed. Samples were analyzed in triplicate, Statistical analysis was performed using Prism version 7.04 (GraphPad Software). s

### 2.7 Targeted multiple reaction monitoring lipid profiling

To validate the involvement of lipids in ARSACS pathogenesis we performed lipid quali/quantitative analysis by LC-MS. Cell lipid extracts were obtained, using a modified Folch approach (Folch et al., 1957), from fibroblasts derived from the same individuals who underwent proteomics analysis (Pt 1-6 and three age-/sex-matched healthy controls). Samples were analyzed in triplicate. In brief, 60 μL of cell extract was diluted with 90 μL of H_2_O, after which 1.5 mL of MeOH/CHCl_3_ (dilution ratio 1:2) was added, and the solution was mixed and left at room temperature for 10 min. Then, 300 μL of 150 mM NaCl aqueous solution was added and the biphasic solution thus formed was incubated at 4°C for 30 min at 3,000 rpm in a Microcentrifuge Heraeus Biofuge Fresco (Thermo Fisher Scientific). Removing the upper phase, the lower phase was dried under vacuum at 36°C (Savant Instruments Inc., Farmingdale, NY), resuspended in 80 μL of MeOH 0.1% HCOOH, and transferred to a glass vial for LC-MS/MS analysis. Targeted lipidomic analysis was carried out using the liquid chromatography-electrospray ionization-tandem mass spectrometry (Michelucci et al., 2021). LC-MS/MS analyses were performed using a Nexera X2 HPLC system (Shimadzu, Kyoto, Japan) combined with a QTrap 5500 mass spectrometer (AB Sciex) equipped with an ion source for electrospray. Selected data acquisition was accomplished for 121 lipid species using the Scheduled MRM Algorithm in Analyst Software 1.6.3 (AB Sciex) with a fixed cycle time of 1.5 s. The phosphatidylcholine (PC), lyso-phosphatidylcholine (LPC), phosphatidylethanolamine (PE), lyso-phosphatidylethanolamine (LPE), sphingomyelin (SM), ceramide (Cer), and diacylglycerol (DG) lipid classes were analyzed using the MRM method. MultiQuant 2.1 software (AB Sciex) was used for comparative lipid quantification (see Supplementary Table 2 for details).

### 2.8 Lipidomic data analysis

Lipidomic data were analyzed using the bioinformatic *lipidr* package (version 2.3.3) (Mohamed et al., 2020). Raw data were loaded on *lipidr* and, after quality control and visualization, were normalized using the probabilistic quotient normalization (PQN) method (Dieterle et al., 2006). We performed a supervised multivariate analysis using orthogonal partial least-squares discriminant analysis (OPLS-DA) (Trygg and Wold, 2002) to discriminate lipid sets between conditions (healthy controls and ARSACS patients); the 10 top prioritized lipid classes were investigated, and differential analysis was conducted by comparing lipid profile levels in patients and controls. Differentially produced lipids were considered significant at *p* < 0.05 and with a FC ≥ 1.5. All classes were submitted to lipid set enrichment analysis (LSEA) (Subramanian et al., 2005; Mohamed et al., 2020) to detect preferential enrichment of certain lipid classes in patients versus controls (significance set at *p* < 0.05).

## 3 Results

### 3.1 Proteomics differential expression analysis

**FIGURE 1:**
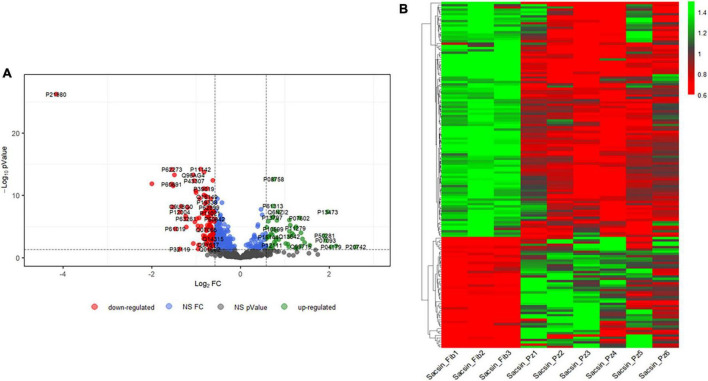
**(A)** Comparison between three control and six ARSACS cell lines. Samples were analyzed in triplicate. Volcano plot showing the differentially expressed proteins (*p*-value threshold = 0.05, log2 FC > | 0.58| corresponding to FC threshold of 1.5 both in up and down-regulation) in ARSACS patients versus healthy controls. Up- and down-regulated proteins are shown in green and red, respectively. UniProt IDs are displayed. NS FC, Non-Significant Fold Change. NS *p*-value, Non-Significant *p*-value. **(B)** Heat map reporting expression levels for the 159 differently expressed proteins in the 9 subjects analyzed (in order, 3 healthy controls and 6 ARSACS patients). For each protein, expression levels are reported as expression values for the single subject normalized on the basis of protein expression mean.

Using the spectral library generated and excluding FDR > 5%, a total of 648 proteins was identified in all individuals (Supplementary Table 3). We identified 159 DEPs: 106 proteins were downregulated, and 53 upregulated in ARSACS fibroblasts with respect to healthy controls, with FC ≥ 1.5 and statistical significance set at *p* < 0.05 (Figure 1A). On observing expression levels of the 159 DEPs (Figure 1B), good intraindividual homogeneity of expression could be seen in each group (Supplementary Figure 1). To categorize a larger number of DEPs for further analyses and limit the effects due to individual variability (Montoro-Gámez et al., 2023), we adopted a less stringent condition (FC ≥ 1.3, *p* < 0.05) and identified a final set of 257 DEPs in ARSACS (Supplementary Figure 2).

### 3.2 Proteomics functional analysis

**FIGURE 2:**
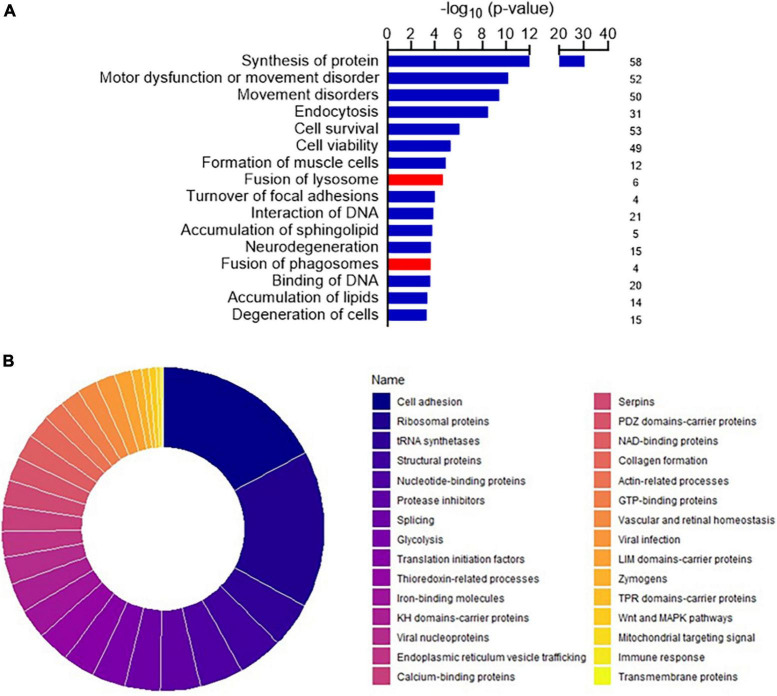
**(A)** Disease and functional annotations exhibiting the most significant impact, along with corresponding activation *z*-scores in the dataset. Red indicates up-regulated protein abundance, while blue signifies down-regulated protein abundance. The histogram shows the numbers of differentially expressed proteins (DEPs) associated with the dysfunctional pathway. **(B)** Donut chart showing the importance in terms of enrichment score of the 30 clusters pinpointed by functional annotation analysis conducted for DEPs using DAVID Bioinformatics Resources. Clusters were ordered according to their respective enrichment scores each cluster groups together annotation terms with similar biological meaning. Enrichment scores were calculated on the basis of the cluster members’ *p*-values. The single clusters are described in detail in Supplementary Figure 6.

**FIGURE 3:**
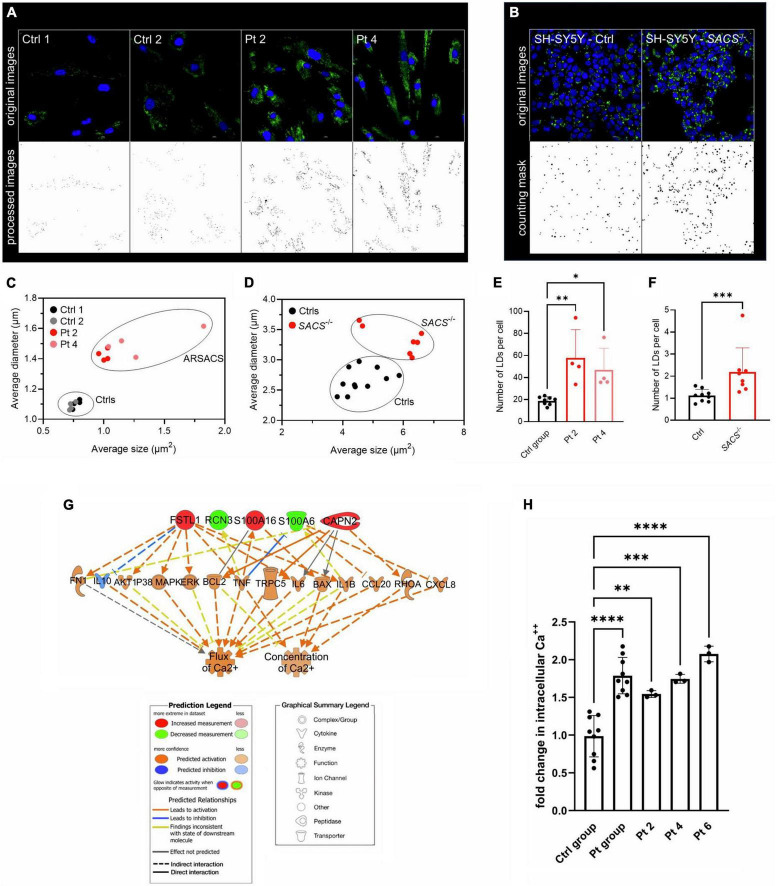
**(A)** Detection of lipid droplets (LDs) in fibroblasts and **(B)** in SH-SY5Y KO cells. Representative images are shown. **(C)** Identification of two morphological clusters based on the average size and diameters in fibroblasts **(D)** and in SH-SY5Y cells. **(E)** LDs count per cell showed a significant increase in ARSACS fibroblasts and in **(F)** and in *SACS*^–/–^ SH-SY5Y cells. SD is shown. One-way ANOVA was performed for statistical analysis in fibroblasts, whereas Mann-Whitney test was carried out for that in SH-SY5Y cells. *p ≤ 0.05, **p ≤ 0.01, ***p ≤ 0.001. At least five fields from four different images of fibroblasts, 800 SACS KO cells (from 7 different fields), and 1,400 control cells (from 10 different fields). Ctrl group, healthy controls (*n* = 2); Samples were analyzed in triplicate. **(G)** Hierarchical representation of the molecular network encompassing DEPs involved in calcium homeostasis. Following a downstream analysis based on experimentally observed causal relationships, we predicted effects of DEPs and molecular nodes on biological functions related to a significant increase in intracellular calcium flux and concentration. **(H)** Analysis of intracellular Ca^2+^ levels in fibroblasts. SD is shown. One-way ANOVA was performed for statistical analysis. **p ≤ 0.01, ***p ≤ 0.001, ****p ≤ 0.0001. Ctrl group, healthy controls (*n* = 3); Pt group, ARSACS patients (*n* = 3). Pt 2-4-6, patients plotted individually. Samples were analyzed in triplicate.

Among the 257 DEPs in the final set, changes in biological states indicated significant impairment in several macro-categories, including *Neurological disorders*, *Cellular Functions and Maintenance*, *Protein Synthesis*, and *Lipid Metabolism* (Supplementary Table 4). Bioinformatic categorization by IPA highlighted dysregulated functional annotations leading to *Accumulation of lipid* (*p*-value 4.34 E-04; z-score −1.508) and *Accumulation of sphingolipid* (*p*-value 1.67 E-04; z-score −1.956) (Figure 2A). Annotations related to the accumulation of gangliosides and glucosylceramides were not possible with IPA because of the relatively low levels of detection of the involved DEPs. Accumulation of lipids was functionally validated by lipid droplets detection in both ARSACS fibroblasts and SH-SY5Y knock-out cells (Figures 3A–F). GO enrichment analysis highlighted significant involvement of *Metabolic processes* (e.g., RNA metabolic processes, protein transport and localization, immune response), *RNA binding and cell adhesion molecules*, and *Extracellular vesicles, exosomes, extracellular space components, ribosomes, ribonucleoprotein complexes* in the BPs, MFs, and CCs categories, respectively (Supplementary Figures 3–5). Finally, a comprehensive functional annotation analysis performed using DAVID pinpointed 30 clusters (Figure 2B), each consisting of enriched annotation terms with similar biological meaning (Supplementary Figure 6). Our analysis unveiled both known and novel biological processes potentially involved in ARSACS. Of note, in a novel development with respect to our previous organelle-proteomics studies (Morani et al., 2022), cluster analysis predicted the involvement of calcium homeostasis in ARSACS pathogenesis by defining dysregulation of several calcium binding proteins (*Calcium-binding proteins* cluster; Figure 2B). In line with the recent robust implication of calcium metabolism in the degeneration of *Sacs*^–/–^ Purkinje cells (Del Bondio et al., 2023), using IPA tools we were able to bioinformatically predict a molecular network that connected DEPs and protein nodes, and predicts an increased calcium flux and concentration (Figure 3G). The latter was functionally validated by the finding of significantly increased intracellular calcium levels in ARSACS fibroblasts (Figure 3H).

### 3.3 Differential lipidomic analysis

Analysis and comparative quantification of lipids (targeted differential lipidomics) was conducted on 121 lipid species. First, relative abundance of lipids in cell extracts was determined by LC-MS analysis using MRM. This technique, already described by us (Michelucci et al., 2021) allows direct quantification of lipid species by specifically comparing MS transitions of 121 lipid species. OPLS-DA plot (Supplementary Figure 7A) showed a good separation between groups and, in line with what others have already observed (Worley and Powers, 2016), the greater the distance between the groups, the higher the discriminating power found in our lipid analysis. The R2X and R2Y factors of the OPLS-DA model were 0.61 and 0.97, respectively, indicating that 61% of the lipid species-level variation and 97% of the group variation could be explained by the model. *lipidr* and OPLS-DA allowed us to define the top ten classes of lipids contributing to differentiate ARSACS from controls, namely PE (40:4), PE (40:5), PC (34:1), DG (32:0), PC (40:2), LPE (18:0), LPC (16:0), LPC (18:1), LPC (20:3), and SM (41:2) (Supplementary Figure 7B).

**FIGURE 4:**
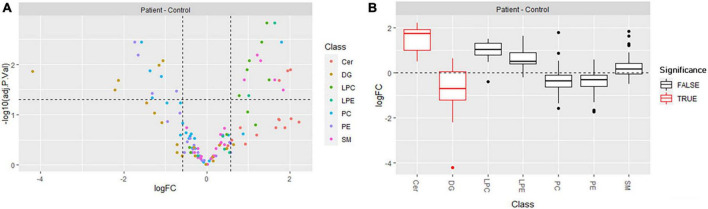
**(A)** Volcano plot showing the differentially produced lipids (*p*-value threshold = 0.05, fold change threshold ≥ 1.5) in ARSACS patients versus healthy controls. Lipid species are colored according to lipid class (Cer, DG, LPC, LPE, PC, PE, SM). **(B)** Distribution of log2 fold change (logFC) per lipid class, with lipid set enrichment analysis results. Significantly enriched classes, Cer and DG, are shown in red.

To assess differentially produced lipids, we conducted univariate analysis (FC ≥ 1.5, with statistical significance set at *p* < 0.05) and found that 13 lipids were downregulated and 17 upregulated in ARSACS with respect to controls (Figure 4A; Supplementary Table 5). Blocks of differentially regulated lipids could easily be distinguished between the two groups: lipid species belonging to the DG [DG (30:0), DG (32:0), DG (36:3), DG (36:4), DG (40:6)], PE [PE (36:1), PE (40:4), PE (40:5), PE (40:6)], and PC [PC (34:1), PC (36:1), PC (36:2), PC (38:4), except PC (40:2)] classes were downregulated, whereas lipid species belonging to the LPE [LPE (18:0), LPE (18:1)], LPC [LPC (16:0), LPC (16:0e), LPC (18:0), LPC (18:1), LPC (20:3), LPC (22:5)], Cer [Cer (d18:0/24:1), Cer (d18:1/24:1), Cer (d18:2/23:1)], and SM [SM (37:1), SM (41:2), SM (41:3), SM (43:2), SM (43:3)] classes were upregulated. Finally, all lipids were submitted to LSEA, a computational method for determining whether an a priori set of lipids shows concordant and statistically significant differences between biological conditions. After lipids were ranked by their FCs, enrichment scores and significance were calculated using an efficient permutation algorithm. Positive and negative enrichment scores indicated up- or downregulation of lipid classes between patients and controls. The distributions of the log2 FC (logFC) values of lipid molecules belonging to each class showed that ceramides were preferentially up-regulated, and lipids belonging to the DG class were preferentially down-regulated, whereas the other classes of lipids did not vary significantly between groups (Figure 4B).

## 4 Discussion

Leveraging on previous omics studies conducted using cell models (Morani et al., 2019, 2021, 2022), we performed untargeted proteomics in fibroblasts from ARSACS patients with different disease durations, severity, and genotypes, with the aim of shedding further light on the pathological role of sacsin in this condition and defining specific disease mechanisms and putative targets for trial readiness. Our proteomics in fibroblasts strengthened the suggestion of involvement of biological processes already associated with ARSACS (such as cell adhesion, vesicle trafficking, autophagy, cell viability) by us and others and confirmed the involvement of lipids and calcium in conditions characterized by loss of sacsin (Stevens et al., 2013; Morani et al., 2019).

Among the most significant dysregulated pathways identified in our analysis, the role of cell adhesion and vesicle trafficking has been only recently highlighted in *SACS*^–/–^ cell and mouse model in which sacsin deficiency drove to altered focal adhesion structure and dynamics with implication in synapses and axons development (Romano et al., 2022). Moreover, our proteomic analysis showed altered expression of several GTP-binding proteins including Rab proteins that have a role in vesicle trafficking, among others, as similarly described by Romano et al. (2022).

Cell viability, autophagy, and oxidative stress have been extensively described by our group and others (Girard et al., 2012; Duncan et al., 2017; Morani et al., 2019), and represent molecular pathways linked to abnormal lipid and calcium homeostasis as discussed below. Our study also confirms recent findings about the involvement of proteins related to movement disorders or encoded by genes known to be mutated in other forms of neurodegeneration (Morani et al., 2021, 2022).

Furthermore, and alike the present study, expression of several ribosomal proteins and those involved in protein synthesis was found also to be altered (Morani et al., 2022), but the link between loss of sacsin and such a fundamental cellular process remains unclear. Similarly, our results indicated an aberrant expression on several tRNA synthetases, whose link with ARSACS is not intuitive, but whose involvement in forms of hereditary ataxia and spastic paraplegia is already known (Antonellis and Green, 2008).

Cytoskeleton components are yet known to have an important role in ARSACS pathogenesis (Duncan et al., 2017; Louit et al., 2023), but microfilaments structure and dynamics have never been reported in ARSACS models. Our analysis indicated an aberrant expression of actin and actin-binding proteins, unraveling a potential role of actin cytoskeleton in the disease. Furthermore, also few structural proteins were found to be dysregulated. Among these, expression of plectin, that acts as linker between intermediate filaments network and other cytoskeletal structures (Winter and Wiche, 2013), was also reduced in ARSACS fibroblasts and SH-SY5Y *SACS*^–/–^ in an independent study (Del Bondio et al., 2023).

Adding to the list of dysregulated pathways, we observed conditions like DNA binding, glycolysis, iron-binding proteins, collagen formation, and innate immune response that remain unclear in the disease scenario.

Calcium is one of the most ubiquitous signaling messengers in the brain, and its relationship with mitochondria and oxidative stress is a common issue in neurodegeneration (Schrank et al., 2020), as well as in hereditary ataxias and spastic paraplegias (Robinson et al., 2020). A recent study in *Sacs*^–/–^ mice proved that abnormal calcium homeostasis has an impact on Purkinje cell degeneration as result of impaired mitochondria and ER trafficking to distal dendrites, and that severe down-regulation of key Ca^2+^ buffer proteins is implicated in disease severity (Del Bondio et al., 2023). Our results corroborated these findings in a patient-derived model, and also supported a potential link with enhanced mitochondrial and cytosolic ROS production in disease status (Baev et al., 2022). Furthermore, the impaired levels of ceramides and DGs that we observed might also relate to calcium sensing through the stimulation of lipid peroxidation, phospholipase C activation, and consequent IP3 production, the latter leading to further impairment of calcium signals. Also, ceramides, known to influence to ROS production and signaling (Dumitru et al., 2007), can induce cell death in cultured cells by a mechanism involving impaired Ca^2+^ influx, mitochondrial network fragmentation, and loss of mitochondrial Ca^2+^ buffering capacity (Parra et al., 2013). On the other hand, DGs are lipid second messengers generated in response to extracellular stimuli and channel intracellular signals that affect mammalian cell proliferation, survival, and motility. DGs exert a myriad of biological functions through protein kinase C, whose function is strictly related to calcium intracellular flux, given that its activation requires binding of Ca^2+^. It is therefore reasonable to hypothesize that the increased intracellular Ca^2+^ levels observed in sacsin-deficient cells might somehow be related to low levels of DGs. Together, the findings of our study raise the novel suggestion that impaired lipid metabolism may be linked to energy dysfunction, calcium homeostasis, and ROS overproduction in ARSACS. Nonetheless, the mechanistic link between reduced DGs by lipid LC-MS and dysregulated proteins remains to be understood.

The up regulation of ceramides found in our work also puts ARSACS in the same pathogenic category as more common forms of neurodegeneration. Ceramides belong to the sphingolipid family, a group of bioactive lipids with signaling mechanisms involved in the regulation of apoptosis, autophagy, proliferation, and differentiation (reviewed in Hannun and Obeid, 2018). Ceramides are also precursors of more complex sphingolipids like sphingomyelin, the main component of the membranous myelin sheath, and of cerebrosides and gangliosides, abundant in nerve cells (Gault et al., 2010). Dysregulation of ceramide metabolism has been observed in common neurological disorders, including multiple sclerosis and Alzheimer’s disease, where, for instance, ceramides promote aggregation of Aβ through interaction of lipid rafts, and ceramide-enriched exosome membranes (Czubowicz et al., 2019). High levels of ceramides have been found to enhance binding affinity for α-synuclein in Parkinson’s disease, leading to increased α-synuclein accumulation, aggregation, and propagation (Kurzawa-Akanbi et al., 2021). Downstream pathogenetic effects of increased ceramide levels are still unclear, but it is worth noting that supplementation of exogenous ceramides was seen to reduce α-synuclein accumulation and protein ubiquitination (Kim et al., 2018). Ceramides are also known to affect the autophagic flux via Beclin1/Bcl-2 or mTOR and can induce changes in membrane fluidity and membrane trafficking (Young et al., 2013). Also, mutations in ceramide biosynthesis enzymes are a cause of other hereditary neurological disorders resembling ARSACS. For example, pathogenic variants in the ceramide synthase gene *CERS2* (Mosbech et al., 2014) cause epilepsy and ataxia, and mutations in genes involved in sphingolipid metabolism such as *B4GALNT1*, *GBA2*, and even *FA2H*, cause different forms of spasticity and ataxia, such as SPG26 (Wilkinson et al., 2005), SPG46 (Boukhris et al., 2010), and SPG35 (Dick et al., 2008), respectively. A recent lipidomic study in Friedreich ataxia fibroblasts also described enhanced synthesis of several ceramides (Wang et al., 2022).

In our study, proteomics indicated the dysregulation of several protein involved in ceramides homeostasis. Glucosylceramidase beta 1 (GBA1), cathepsin B (CTSB), and prosaposin (PSAP) are tightly linked in this process and are all upregulated in our dataset. GBA1 hydrolyzes glucosylceramide into ceramide and glucose in lysosomes (Schapira, 2015), and a recent study showed that CTSB is activated by ceramides to promote PSAP cleavage to saposin C, a coactivator of GBA1 in lysosomes, and that this process is altered in Parkinson disease (Kim et al., 2022). Hence, we could speculate that upregulation of these proteins boosts the whole pathway increasing of the levels of ceramides when sacsin-is missing. Furthermore, it was newly demonstrated that GTPase Rab14, upregulated in our proteomic dataset, regulates the trafficking of ceramide from endoplasmic reticulum to Golgi apparatus (Liu et al., 2023), corroborating the increased levels of ceramides identified in our study. Interestingly, two upregulated proteins in our dataset seem to be implied in ceramide homeostasis, namely caveolin-1 (CAV1) and superoxide dismutase 2 (SOD2). Caveolin-1 was found to regulate the generation of ceramide-dependent organization of the plasma membrane (Ketteler et al., 2020), whereas an increase of SOD2/SOD1 ratio was activated by ceramide to foster apoptosis (Chang et al., 2018). Finally, lipidomic analysis evidenced significant upregulation of lyso-phospholipids belonging to the family of lyso-PC and lyso-PE. Notably, there is no significant dysregulation of lyso-phospholipids as overall classes. However, the important (logFC: 1.02–1.64) overexpression of lyso-PC and lyso-PE might suggest the onset of cell response linked to the inflammatory pathway. This agrees with the observed downregulation (logFC: 1.31–1.65) of polyunsaturated (4–6 unsaturations) PC and PE. Generation of lyso-PC and lyso-PE has been in fact linked to the action of intracellular ROS (Engel et al., 2021). Polyunsaturated glycerophospholipids have an assessed role in mediating and propagating oxidative stress signaling. Thus, the observed reduction of saturation in PC and PE might suggest that defense mechanisms in the cell occur to minimize the effect of ROS generation.

Limitations of our study include the use of a peripheral tissue not directly affected by neurodegeneration, and an LC-MS lipid quali/quantitative approach, which might have led us to overlook some classes of lipids. However the two classes of lipids identified (ceramides and DGs) present several element of interest relevant to other forms of hereditary spastic ataxia.

To summarize, our proteomics and lipidomic study in fibroblasts from ARSACS patients highlighted altered levels of ceramides and DGs, which may potentially be involved in impaired cell signaling in ARSACS etiopathogenesis. Confirmation of these findings in motor neurons would allow us to speculate on targets for treatments (Maines et al., 2023) or monitor disease severity through surrogates of metabolic status in ARSACS.

## Data availability statement

The datasets presented in this study can be found in online repositories. The names of the repository/repositories and accession number(s) can be found at: ProteomeXchange (via the PRIDE database), Project accession: PXD049199.

## Ethics statement

The studies involving humans were approved by the Comitato Etico Pediatrico Regione Toscana. The studies were conducted in accordance with the local legislation and institutional requirements. The participants provided their written informed consent to participate in this study. Written informed consent was obtained from the individual(s) for the publication of any potentially identifiable images or data included in this article.

## Author contributions

DG: Data curation, Formal analysis, Investigation, Methodology, Validation, Visualization, Writing – original draft, Writing – review & editing. SR: Methodology, Software, Validation, Visualization, Writing – review & editing. ND: Data curation, Methodology, Software, Validation, Visualization, Writing – review & editing. FD: Validation, Writing – review & editing, Writing – original draft. GS: Visualization, Writing – review & editing, Writing – original draft. FM: Writing – review & editing. EC: Data curation, Visualization, Writing – review & editing, Writing – original draft. SD: Conceptualization, Formal analysis, Methodology, Software, Supervision, Validation, Visualization, Writing – review & editing. FS: Conceptualization, Funding acquisition, Project administration, Resources, Supervision, Visualization, Writing – review & editing.

## Footnotes

1
http://www.r-project.it

2
https://cytoscape.org
