# Supplementary material for: Proteomics and lipidomic analysis reveal dysregulated pathways associated with loss of sacsin
Source: Front Neurosci. 2024 Jun 7;18:1375299. doi: 10.3389/fnins.2024.1375299 (PMC11191878; doi:10.3389/fnins.2024.1375299)
Supplement: Supplementary file 2 [file Data_Sheet_2.docx]

Supplementary Material

# Supplementary Figures


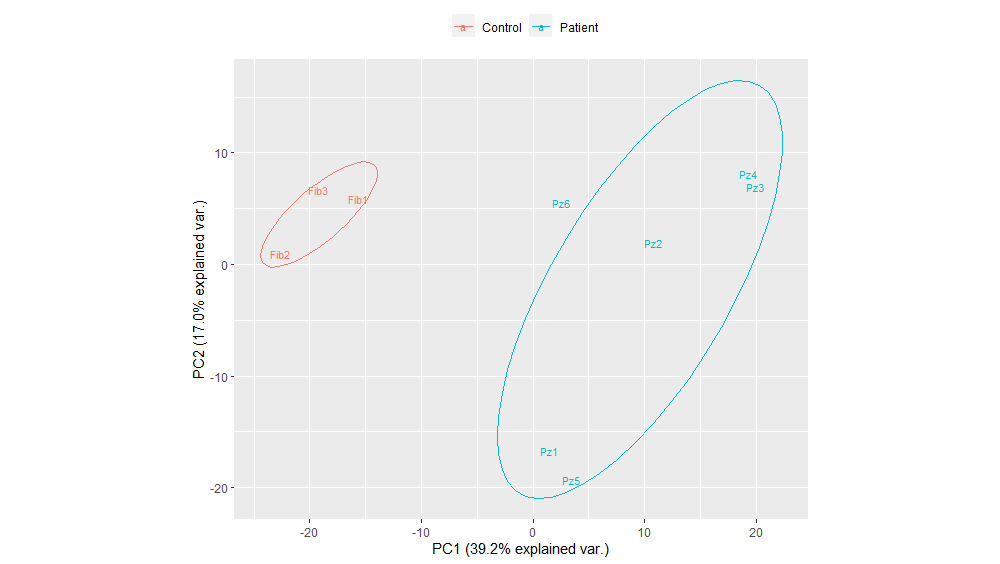


**Figure S1**. Principal Component Analysis (PCA) plot demonstrating the separation between the two groups (three control and six ARSACS cell lines). The variance explained by each variable is shown in brackets.


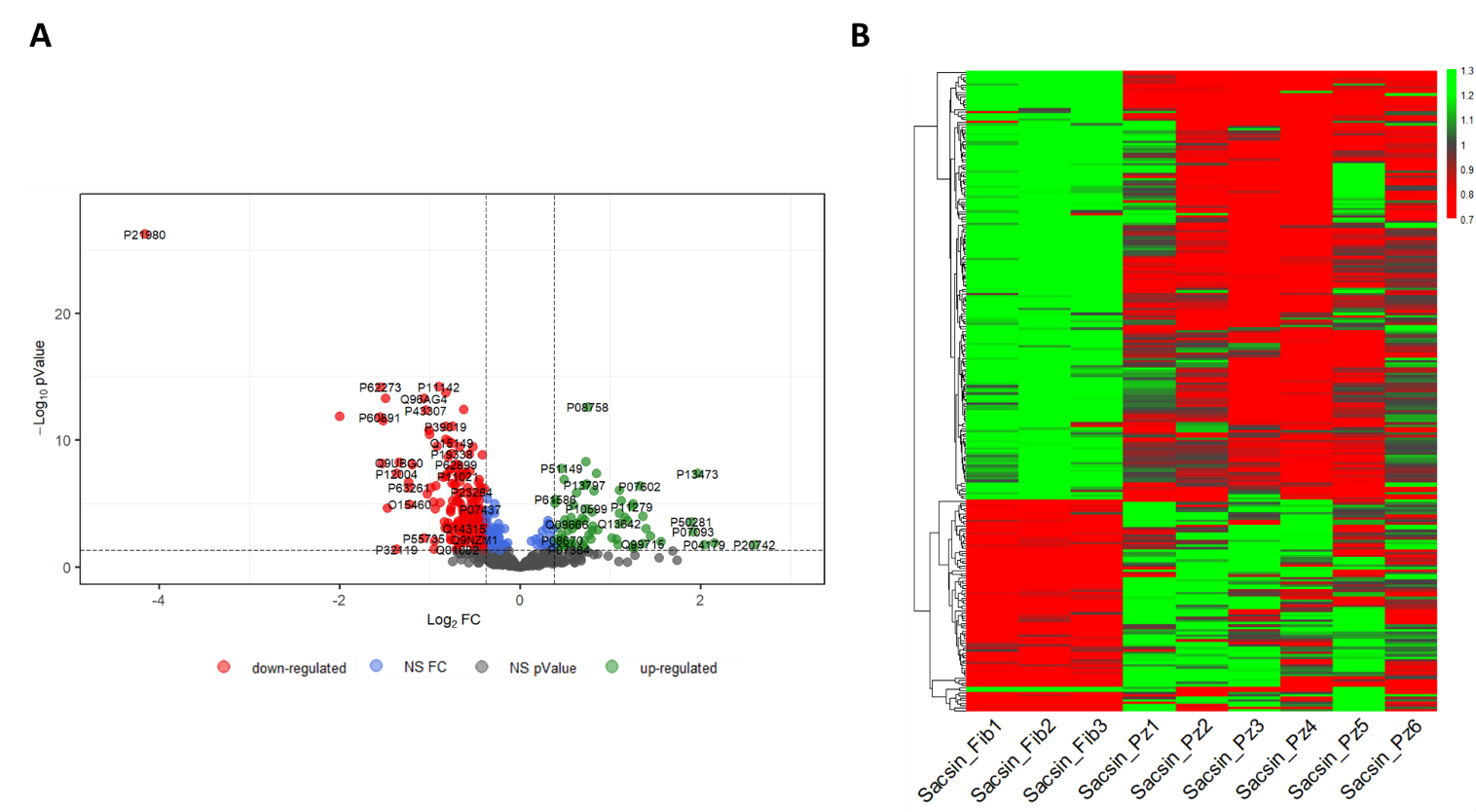


**Figure S2**. (**A**) Comparison between the two groups (three control and six ARSACS cell lines). Samples were analyzed in triplicate. Volcano plot showing the differentially expressed proteins (p value threshold=0.05, log2 FC > |0.38| corresponding to FC threshold of 1.3 both in up or down-regulation) in ARSACS patients versus healthy controls. Up- and down-regulated proteins are shown in green and red, respectively. UniProt IDs are displayed. (**B**) Heat map reporting expression levels for the 257 differently expressed proteins in 3 controls (Fib1, Fib2, Fib3) and 6 patients (Pz1-Pz6). For each protein, expression levels are reported as values for the single subject normalized to the mean of protein expression.

#
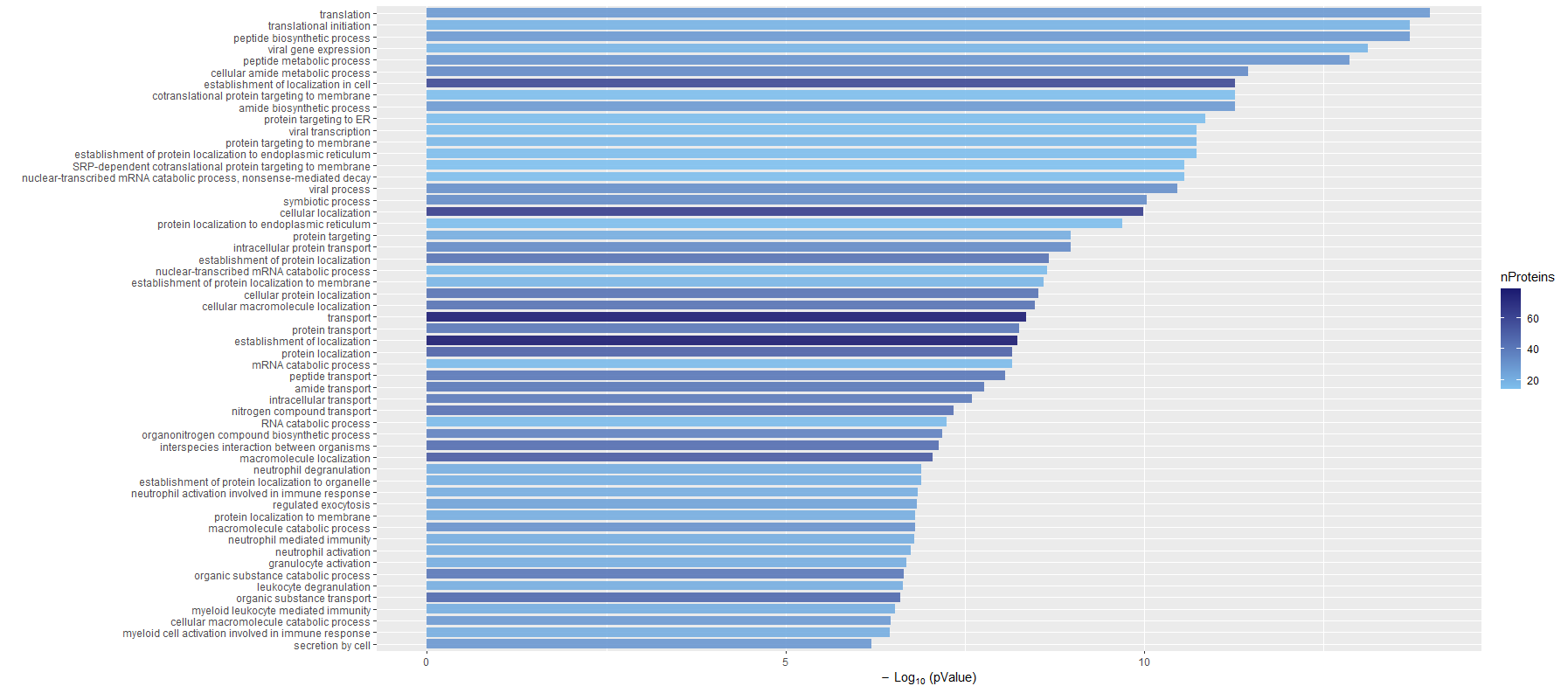


**Figure S3**. GO enrichment analysis results for Biological Processes (BPs) showed significant enrichment of metabolic processes, in particular RNA metabolic processes, protein transport and localization and immune response processes. The bar plot shows the most statistically significant enriched BPs with their relative significance (-log10pValue on X-axis) and their relative number of proteins in the input data (the number of proteins [nProteins] is presented as color scale). We studied two groups (three control and six ARSACS cell lines). Samples were analyzed in triplicate.


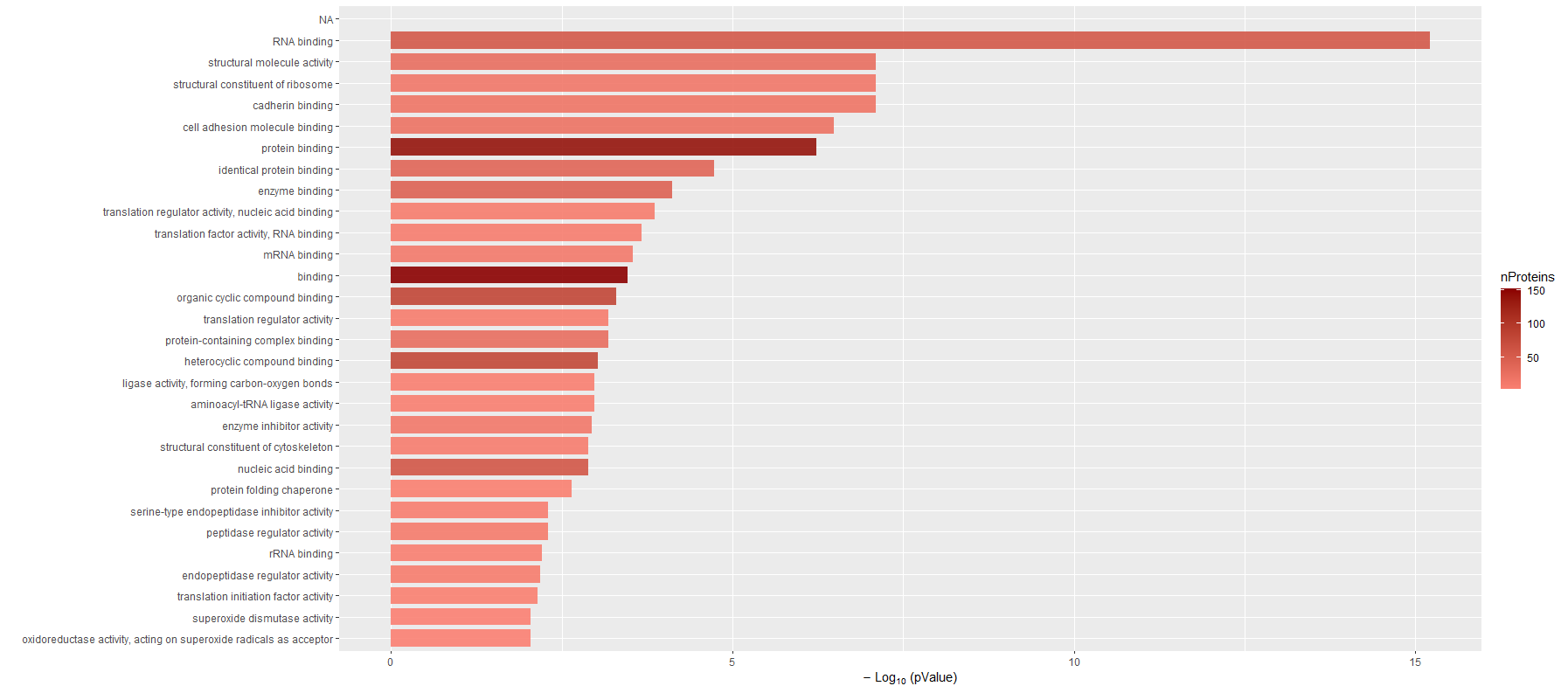


**Figure S4**. GO enrichment analysis results for Molecular Functions (MFs) showed significant enrichment of function associated to RNA binding, structural constituent of ribosome and cell adhesion. The bar plot shows the most statistically significant enriched MFs with their relative significance value (-log10pValue on X-axis) and the relative number of proteins in the input data (the number of proteins [nProteins] is presented as color scale). We studied two groups (three control and six ARSACS cell lines). Samples were analyzed in triplicate.


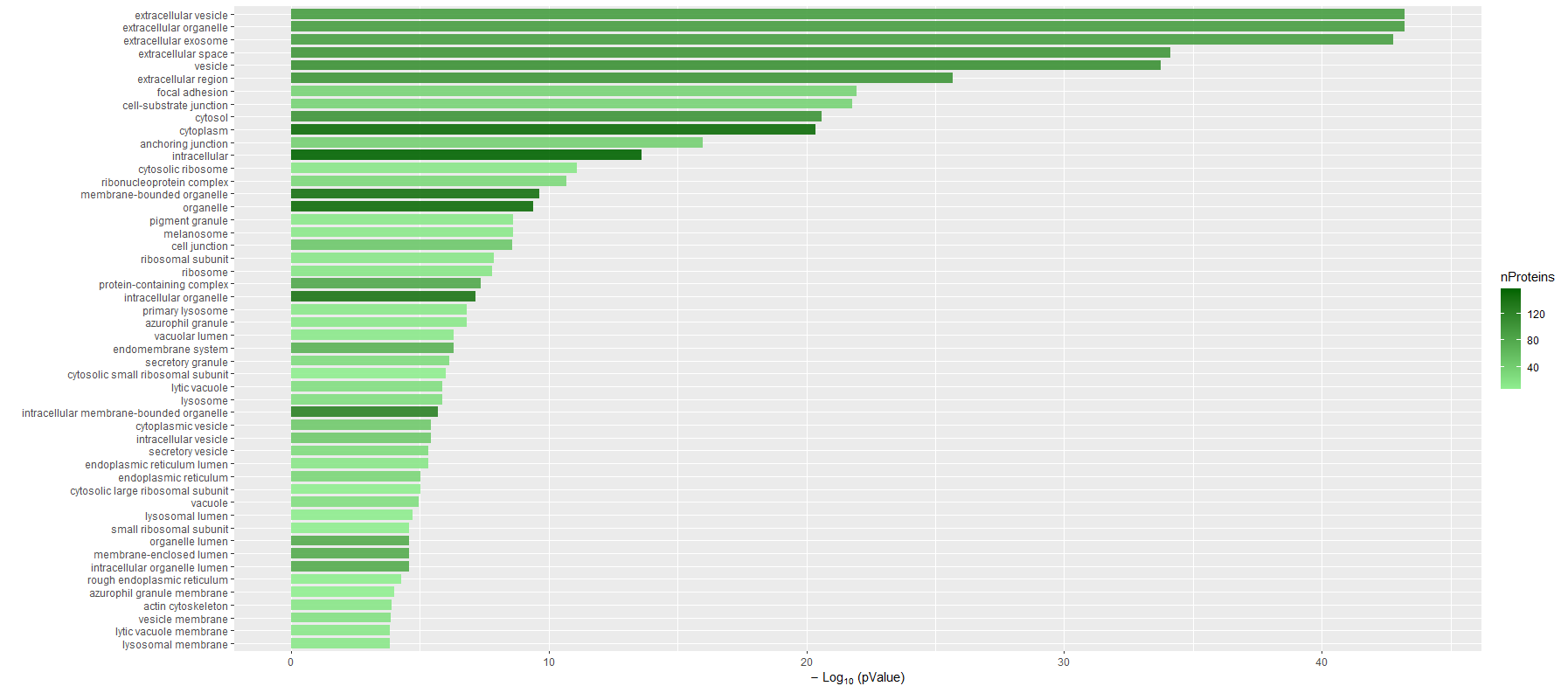


**Figure S5**. GO enrichment analysis results for Cellular Components (CCs) showed significant enrichment of extracellular vesicle, exosome and extracellular space components, ribosome and ribonucleoprotein complex. The bar plot shows the most statistically significant enriched CCs with their relative significance (-log10pValue on X-axis) and their relative number of proteins in the input data (the number of proteins [nProteins] is presented as color scale). We studied two groups (three control and six ARSACS cell lines). Samples were analyzed in triplicate.


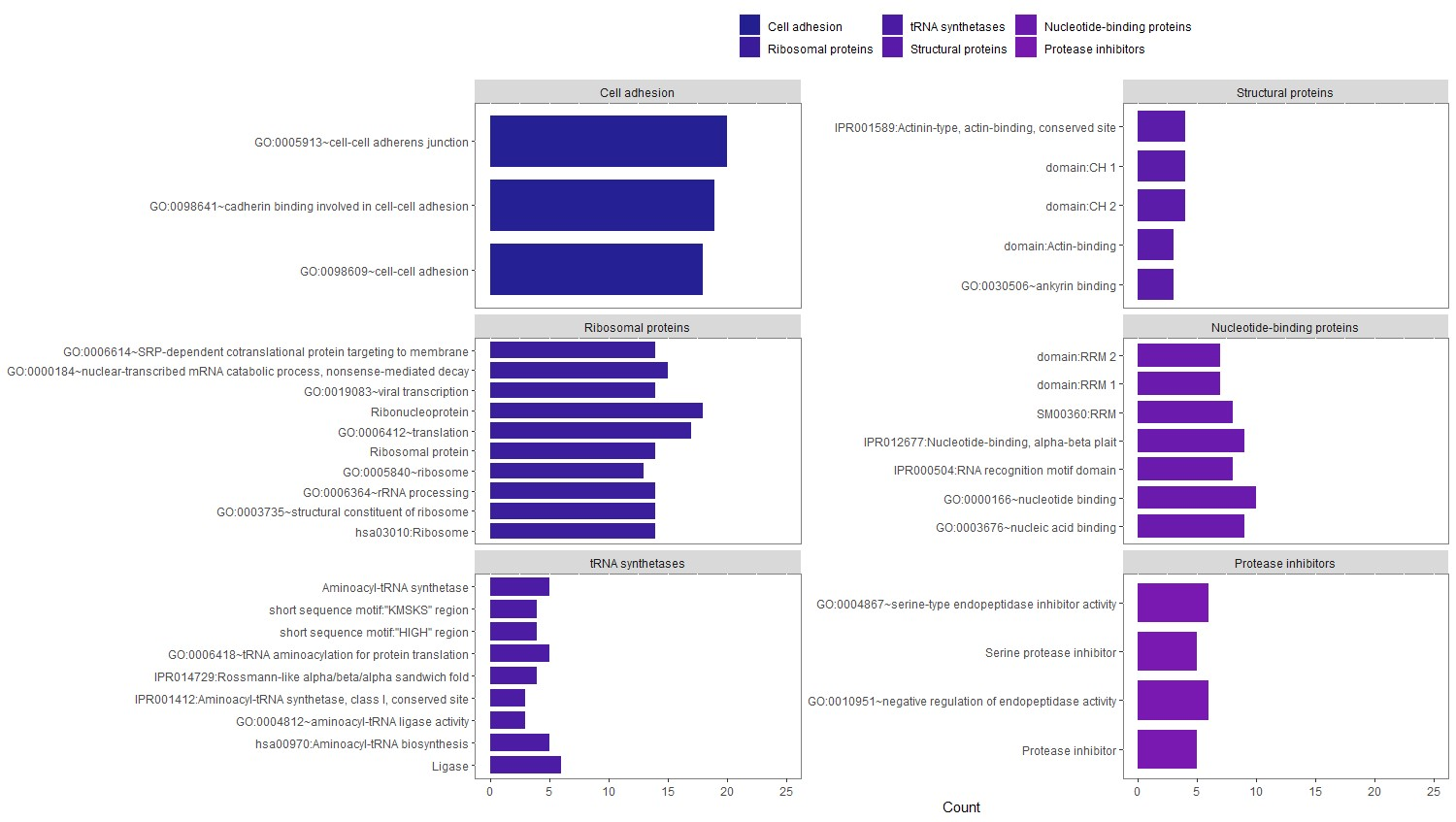


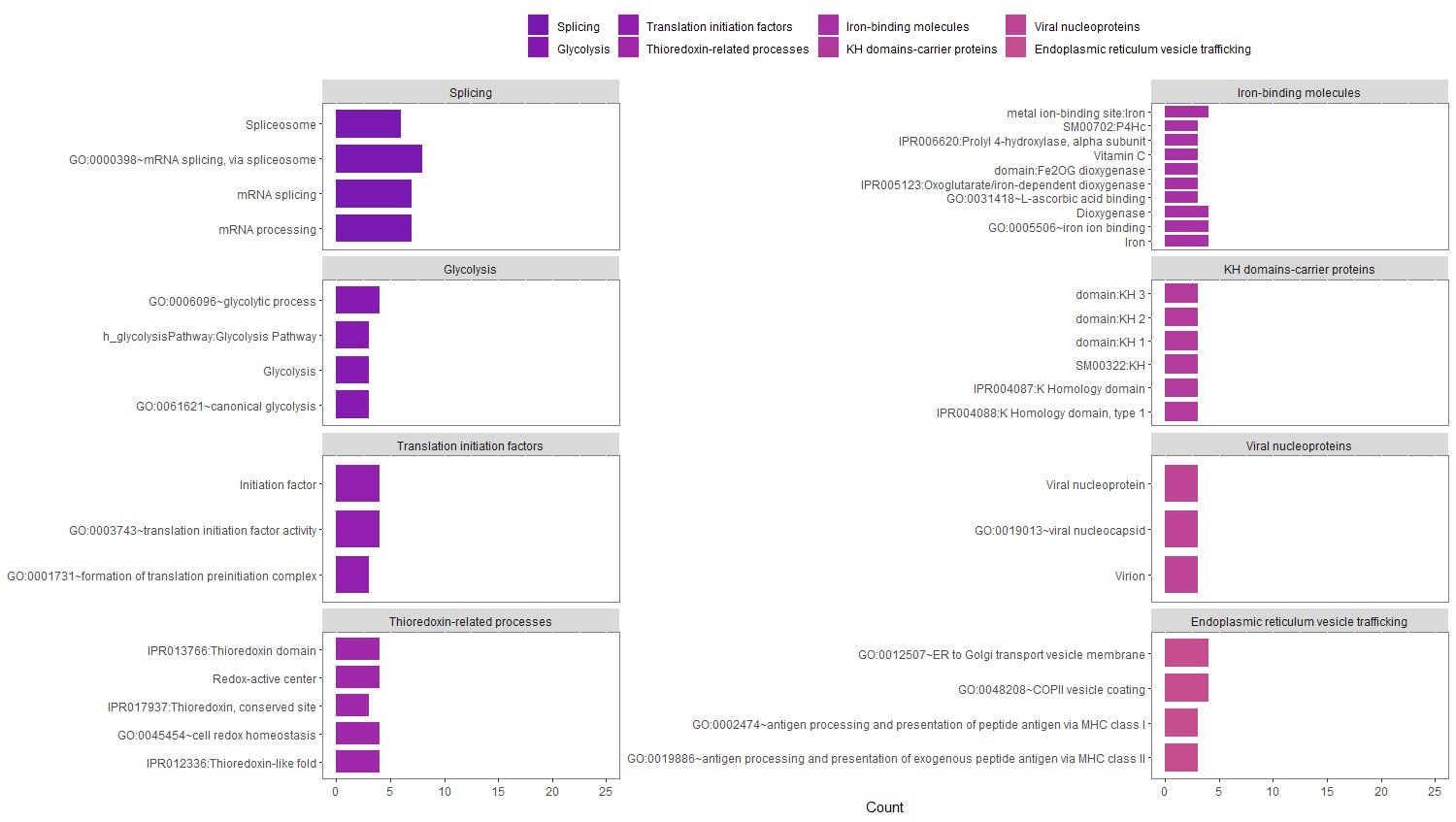


**Figure S6** (continue)


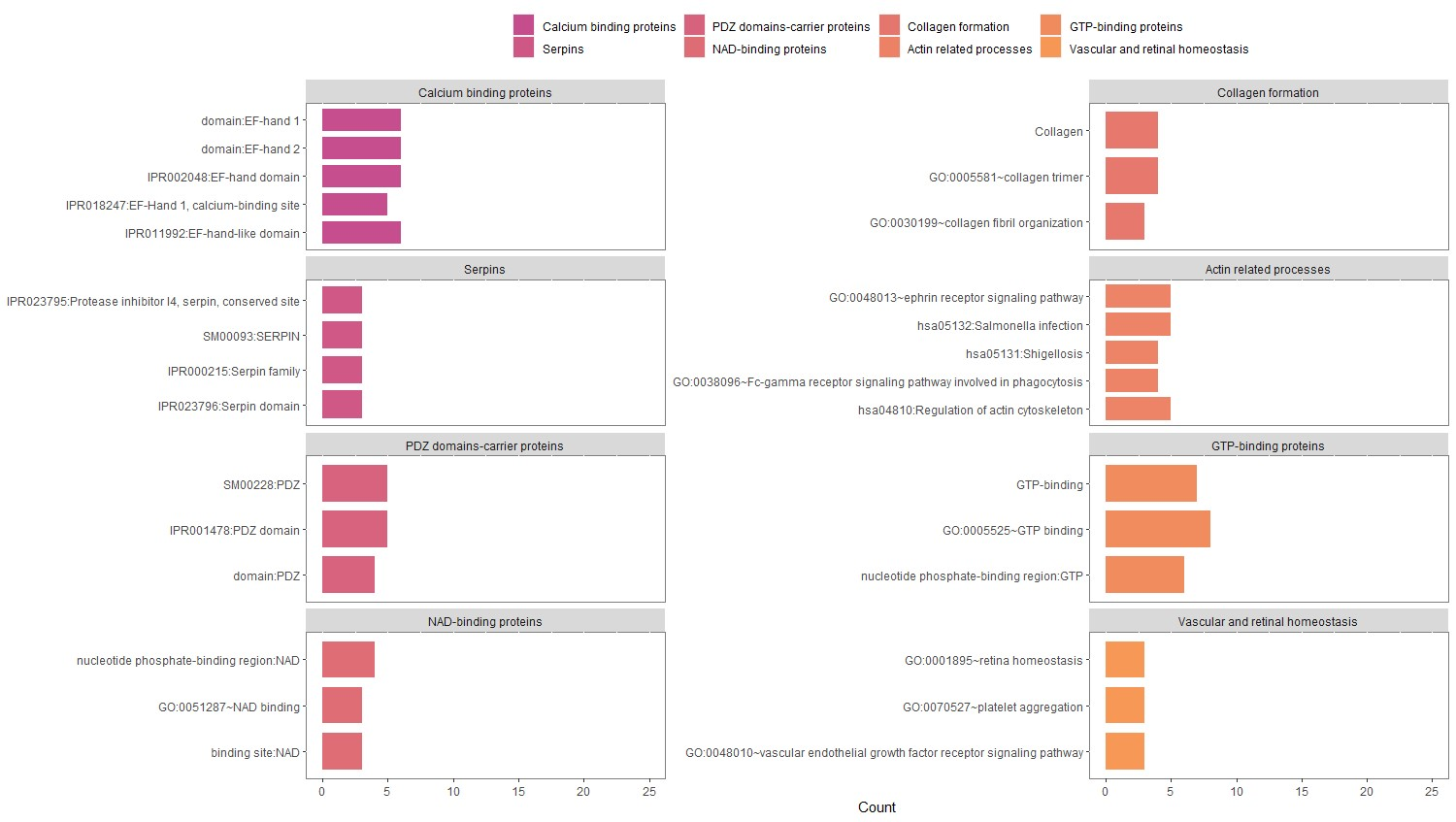


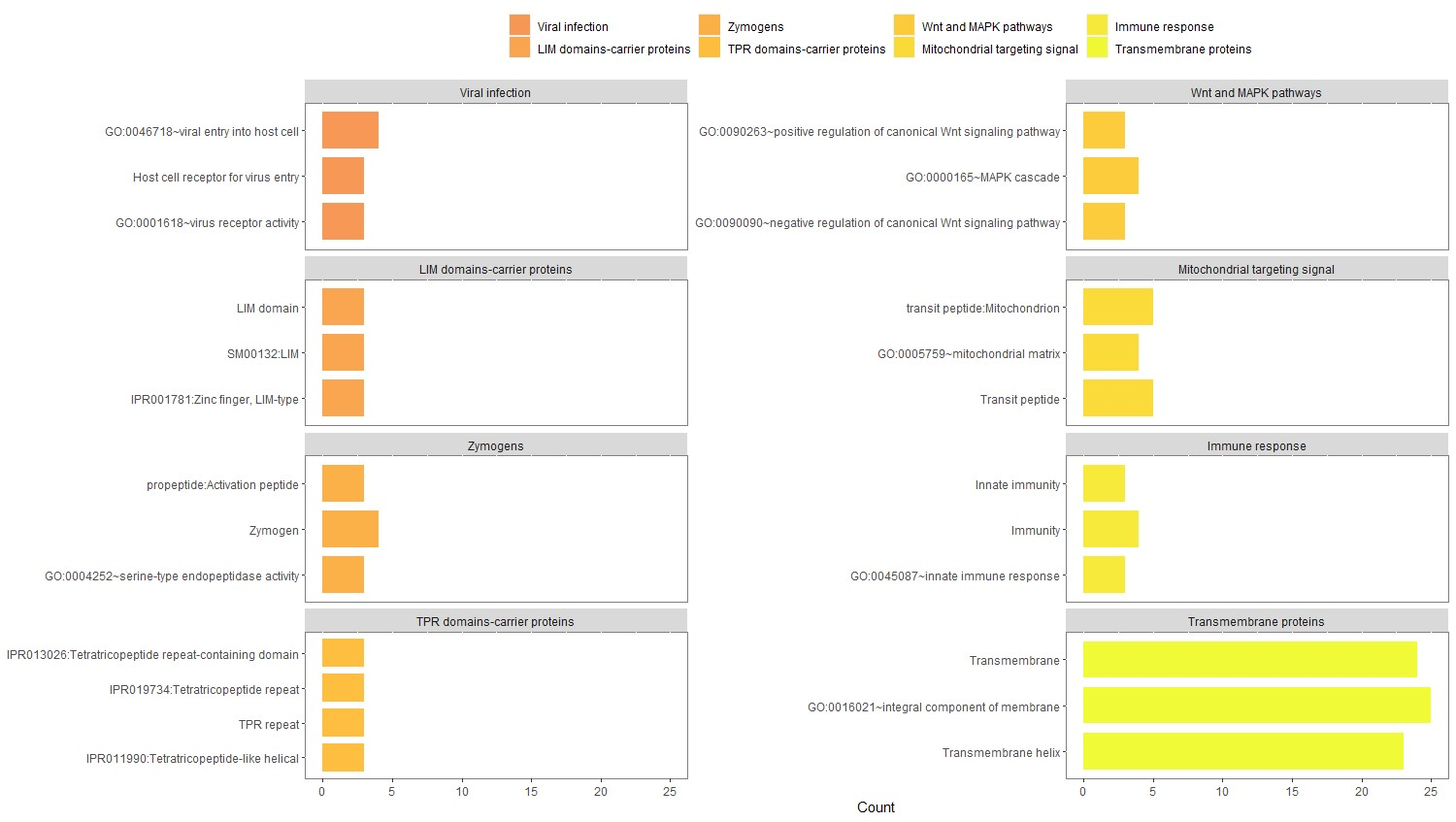


**Figure S6**. Bar plots displaying member terms for the 30 clusters resulted from Functional Annotation Analysis conducted for differentially expressed proteins using DAVID Bioinformatics Resources Functional Annotation Analysis. Each cluster member’s is reported with its relative number of proteins in the input data (Count). We studied two groups (three control and six ARSACS cell lines). Samples were analyzed in triplicate.

**A**


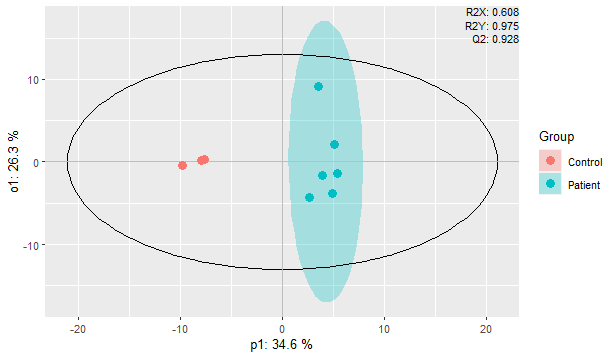


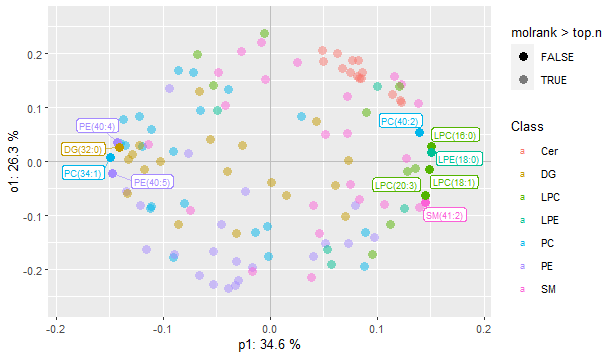

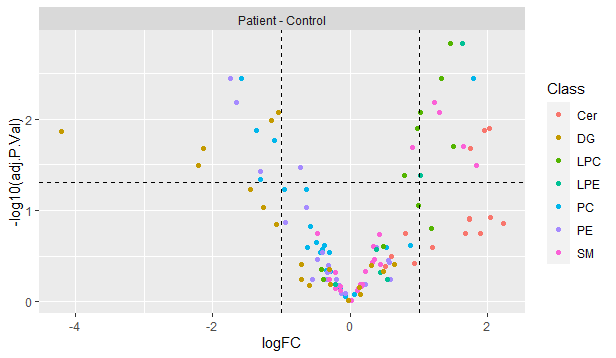


**B**

**Figure S7**. (**A**) OPLS-DA plot for 121 lipid species investigated in differential lipidomic analysis. Discriminant analysis was performed using presence or absence of ARSACS disease as a grouping variable (R2X=0.608, R2Y=0.975, Q2=0.928). Ellipse around patients group shows the group’s 95% confidence region. (**B**) Loadings plot highlights top 10 lipids contributing to the separation between different groups. We studied two groups (three control and six ARSACS cell lines). Samples were analyzed in triplicate.
